# The Impact of the COVID-19 Pandemic on Mortality Rates From Non-Communicable Chronic Diseases in Taiwan: An Interventional Time Series Study

**DOI:** 10.3389/ijph.2025.1607723

**Published:** 2025-03-28

**Authors:** Chen-Mao Liao, Yi-Wei Kao, Chih-Ming Lin, Pei-Yu Lai

**Affiliations:** ^1^ Department of Applied Statistics and Information Science, Ming Chuan University, Taoyuan, Taiwan; ^2^ Department of Healthcare Information and Management, Ming Chuan University, Taoyuan, Taiwan

**Keywords:** COVID-19, mortality, non-communicable disease, time series analysis, healthcare access

## Abstract

**Objectives:**

To examine whether the likelihood of death from non-communicable diseases nationwide was heightened during the COVID-19 pandemic.

**Methods:**

Data on mortality caused by seven leading non-communicable chronic diseases from 2011 to 2022 were extracted from Taiwan’s Death Registry. Monthly standardized mortality rates were analyzed using an intervention time series model.

**Results:**

The monthly mortality rate showed a significant upward trend during the pandemic in the rate of mortality due to heart diseases, diabetes, and hypertension diseases (p < 0.001). The 2021 monthly rates of mortality caused by the three diseases showed a significant increase of 4.3%, 8.2%, and 13.4%, respectively, compared to the 2020 rates and continued until the end of the study period. No upward or downward post-intervention shift was observed for malignant tumors, renal disease, and liver diseases.

**Conclusion:**

Adverse individual behaviors and reduced health services might have raised severe concerns for patients with cardiovascular diseases and diabetes. Health promotion and medical resource allocation are crucial for patients with disadvantaged health and sociodemographic factors and related metabolic conditions during the pandemic.

## Introduction

In March 2020, the WHO declared COVID-19 a global pandemic. By December 2022, over 750 million cases and 6 million deaths were confirmed [1]. WHO estimated 14.83 million excess deaths in 2020–2021, 2.74 times the reported number [2]. Cardiovascular disease and cancer remain the leading causes of death globally [3]. In the USA, two-thirds of excess deaths are attributed to COVID-19 [4]. Deaths from overdose, homicide, and unintentional injuries also rose [5–7]. Excess deaths increased in Southern America [8, 9] and Europe [10–13], while control measures reduced some infectious diseases and injuries [14, 15]. High-risk groups for COVID-19 death include those with non-communicable diseases, men, and the elderly [16–18]. COVID-19 patients might have died from worsened cardiovascular diseases [19].

The excess deaths during the pandemic may be due to COVID-19’s lethality and the overloading of medical services, leading to poor care for chronic disease patients [10–12]. Economic and social conditions may have also increased mortality rates. Studying COVID-19’s effects on mortality can inform healthcare responses and administrative measures [20]. However, a meta-analysis found most studies poorly conducted, highlighting the need for better-designed research to understand non-COVID-19-related mortality [21]. Previous studies often failed to consider time trends, seasonal effects, and population structure. A recent study using a time series model found cardiovascular hospitalizations 16.3% lower than expected but acknowledged limitations due to sampling from one city and data recording delays [22].

Domestic COVID-19 spread in Taiwan began sporadically in early 2021, later than most countries. Hospital hesitations impacted patient access. In May 2021, the Taiwan Central Epidemic Command Center (CECC) initiated large-scale epidemic control measures (Level-2 and Level-3 Alerts). Measures included strict border quarantine, reduced outpatient visits, limited patients and visitors, and suspended non-urgent procedures. Hospitals established negative pressure isolation wards, expanded dedicated wards, and activated contingency hospitals for COVID-19 patients [23]. These measures reduced medical service capacity. COVID-19 cases surged in April 2022, with daily cases exceeding 80,000 by June. Daily deaths surpassed 200, and between 20,000 and 50,000 daily cases persisted until October 2022. In 2022, there were over nine million cases (43% of the population) and 15,755 deaths [24]. This corresponds to approximately 1.7 deaths per 1,000 cases—lower than the global estimate of 5–20 per 1,000. Such a relatively low fatality rate may stem from Taiwan’s robust testing capacity, high healthcare and intensive care accessibility, as well as a strong vaccination campaign. For instance, Taiwan’s high vaccination rates, supported by its National Health Insurance system, have been crucial in managing the pandemic [25]. Additionally, studies have shown that being fully vaccinated against COVID-19 significantly reduces the risk of severe disease and mortality [26]. These factors likely helped reduce severe cases and COVID–19–related mortality.

Some effects of non-pharmaceutical measures on morbidity and mortality during the COVID-19 pandemic in Taiwan have been reported. Gao et al. evaluated if these measures and mass behavioral changes affected mortality rates. They found that the all-cause mortality rate, including deaths from pneumonia and influenza, in 2020 was significantly lower than in 2019, while road traffic accident deaths increased [27]. However, information on mortality from non-communicable causes during the pandemic in Taiwan is limited. This study aimed to examine if the pandemic increased nationwide deaths from specific non-communicable diseases, using a comprehensive timeline of epidemic prevalence and control measures.

## Methods

### Source of Mortality Data

Data on mortality from 2011 to 2022 were extracted from Taiwan’s Death Registry. In Taiwan, physicians determine the cause of death, following the 10th version of the International Classification of Diseases (ICD). Excluding communicable diseases and accidents from the top ten causes of mortality, we counted deaths from leading non-communicable diseases, including malignant neoplasms (ICD = C00–C97), heart diseases (I01–I02.0, I05–I09, I20–I25, I27, I30–I52), cerebrovascular diseases (I60–I69), diabetes mellitus (E10–E14), chronic liver disease and cirrhosis (K70, K73–K74), nephritis, nephrotic syndrome and nephrosis (N00–N07, N17–N19, N25–N27), and hypertensive diseases (I10–I15). Data from death certificates included gender, age at death, and cause of death. We conducted a monthly count of deaths, categorizing them by gender and age. Age-specific populations from Taiwan’s household registrations were used to calculate age-specific mortality rates from 2011 to 2022. The WHO’s 2000–2025 world standard population numbers were used to calculate age-standardized mortality rates. Ethical issues were considered, and the study protocol was approved by the Research Ethics Committee of National Taiwan University (NTU-REC No. 20211EM002).

### Background of the Intervention

In early 2020, Taiwan faced the first wave of COVID-19, with cases mainly from abroad. Early response and stringent quarantine measures blocked the first wave and slowed local spread [28]. However, the second wave in early 2021 caused a sharp surge in cases. To prevent overwhelming medical resources and staff, and to reduce infection risks, the CECC began separating COVID-19 patients into different wards and strengthened the referral mechanism for severe and mild cases starting in May [23].

### Models and Analyses

The research spanned January 2011 to December 2022, covering 12 years with 144 monthly observations of sex-specific mortality rates. Due to decreased hospitalization and stricter hospital access from early 2021, an intervention model based on January 2021 data was established to assess seasonal variations in 120 pre-interventional data points and 24 post-interventional observations. Although some preliminary measures and public awareness emerged in 2020, they were relatively sporadic. Therefore, we chose January 2021 to capture the onset of large-scale outbreak control measures and hospital capacity adjustments. We used an interventional analysis with a noise series following a seasonal autoregressive integrated moving average (SARIMA) model, which is well-suited for evaluating intervention impacts on time series. The autocorrelation and partial autocorrelation functions identified long-term trends and regularities.

An appropriate model for the series’ stochastic behavior was identified, incorporating an interventional component to assess the intervention’s impact on mortality rates over the specified period. The autocorrelation and partial autocorrelation functions were used to identify possible long-term trends and other regularities in the series. Furthermore, the unit root test was performed with an augmented Dickey-Fuller test to identify stationarity for this series. Secondly, a temporary or permanent interventional component was added when an adequate model for the stochastic behavior of the series was identified, resulting in a full impact assessment model. With the standardized monthly mortality rate as the dependent variable, the statistics appropriated intervention models were selected and analyzed as follows.

Let $Y_{t}$ be the standardized monthly mortality rates. The effect can be present with the intervention model as follows.

$$Y_{t}=\frac{\omega_{m}\left(B\right)B^{b}}{\delta_{r}\left(B\right)}S_{t}^{T}+N_{t},$$

where the intervention is the step function,

$$S_{t}^{T}=\left\{\begin{array}{cc} 0, & t<T\\ 1, & t\geq T \end{array}\right..$$

*T* is the beginning of the intervention, $\omega_{m}\left(B\right)=\omega_{0}+\omega_{1}B+\cdots +\omega_{m}B^{m}$ is the initial effects of the intervention, $\delta_{r}\left(B\right)=1-\delta_{1}B-\cdots -\delta_{r}B^{r}$ is the subsequent effect of the intervention, *b* is the time delay of the intervention effect, and *B* is the back shift operator. $\omega_{m}\left(B\right)$ and $\delta_{r}\left(B\right)$ are tested to identify whether the effect of the intervention is temporary, permanent, interrupted, or gradual. The noise process $N_{t}$ is the seasonal ARIMA model (denote by $\left.SARIMA\left[\left(p,d,q\right)\times {\left(P,D,Q\right)}_{s}\right]\right)$ as follows.

$$\phi_{p}\left(B\right){\left(1-B\right)}^{d}\Phi_{P}\left(B^{s}\right){\left(1-B^{s}\right)}^{D}N_{t}=C+\theta_{q}\left(B\right)\Theta_{Q}\left(B^{s}\right)a_{t},$$

where *C* is the drift coefficient, $a_{t}$ is the white noise process, $\phi_{p}\left(B\right)=1-\phi_{1}B-\cdots -\phi_{p}B^{p}$ and $\theta_{q}\left(B\right)=1+\theta_{q}B+\cdots +\theta_{q}B^{q}$ are the regular autoregressive and moving average factors, $\Phi_{P}\left(B\right)=1-\Phi_{1}B^{s}-\cdots -\Phi_{P}B^{sP}$ and $\Theta_{Q}\left(B\right)=1+\Theta_{1}B^{s}+\cdots +\Theta_{Q}B^{sQ}$ are seasonal autoregressive and moving average factors for *s* seasonal period, and ${\left(1-B\right)}^{d}$ and ${\left(1-B^{s}\right)}^{D}$ are the differencing factors for the regular and seasonal periods, respectively.

The interventional components were also examined during the months of the intervention and the first to the twelfth month before and after the intervention. The potential lack-of-fit tests of these models were determined using Box–Ljung statistics [29, 30]. Finally, the appropriate intervention models were selected using Akaike’s information criterion based on the maximum likelihood method. SAS version 9.1 (SAS Institute Inc., Cary, NC, United States) was used for storage and aggregation of the data, and all SRIMA analyses were performed with R version 3.2.5 (R Foundation for Statistics Computing, Vienna, Austria)

## Results

**FIGURE 1:**
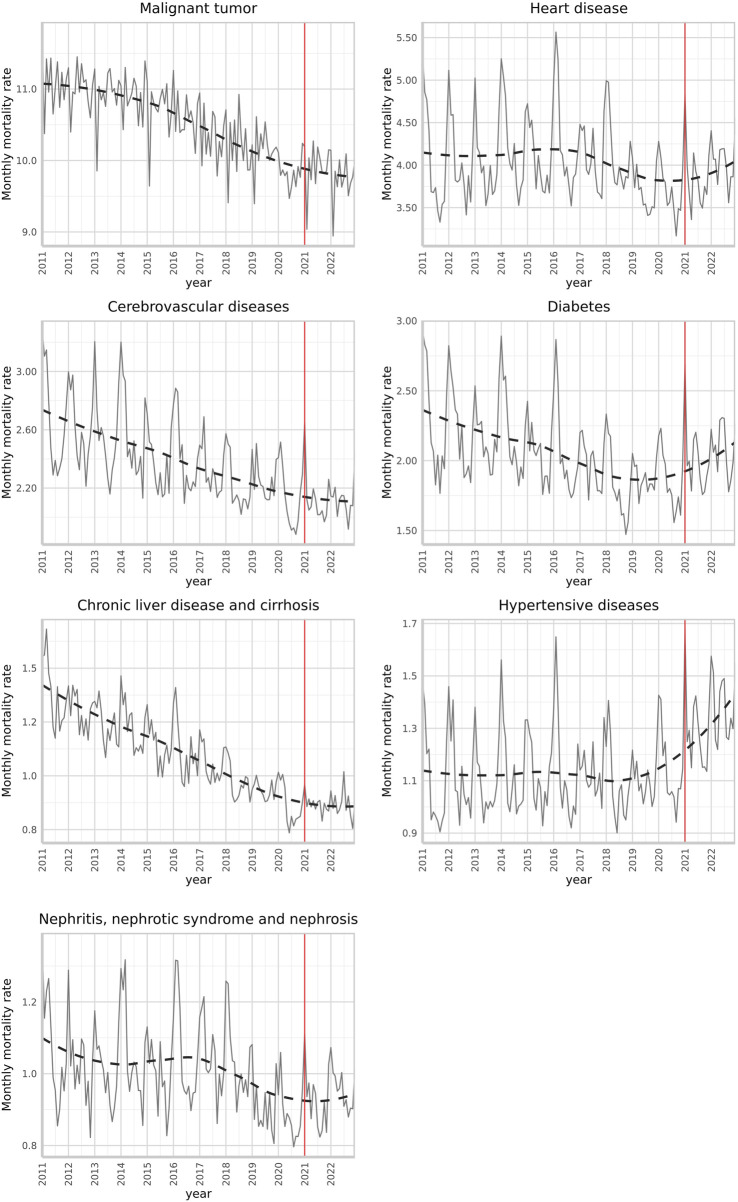
Standardized monthly mortality rates (per 100,000 persons) due to leading non-communicable diseases and a one-step-ahead forecast with an intervention began in January 2021 (Taiwan, 2011–2022). (The Impact of the COVID-19 Pandemicon Mortality Rates From Non-Communicable Chronic Diseases in Taiwan: An InterventionalTimeSeriesStudy.Taiwan. 2011-2022).

Table 1 shows an oscillating trend of crude mortality for non-communicable diseases. Malignant tumors had the highest mortality rate (about 200 per 100,000) over the past decade. However, this trend diminished when age-standardized, especially for malignant tumors, cerebrovascular diseases, diabetes, and liver diseases (Figure 1; Table 2). Notably, mortality increases were apparent in January and February, the coldest months in Taiwan, which typically worsen disease severity. The 12-year monthly rate and one-step-ahead forecast showed trend differences after the January 2021 intervention (Figure 1). We observed an upward trend in heart diseases, diabetes, and hypertension mortality rates post-intervention. No abrupt post-intervention decrease was observed for the other diseases, with liver diseases showing relative stability.

**TABLE 1:** Crude annual mortality rates (per 100,000 persons) due to leading non-communicable diseases (Taiwan, 2011–2022). (The Impact of the COVID-19 Pandemicon Mortality Rates From Non-Communicable Chronic Diseases in Taiwan: An InterventionalTimeSeriesStudy.Taiwan. 2011-2022).

Diseases	2011	2012	2013	2014	2015	2016	2017	2018	2019	2020	2021	2022
Malignant tumor	183.50	187.64	191.87	196.95	199.59	203.10	203.93	206.89	212.88	212.71	220.11	222.67
Heart disease	71.20	73.57	75.79	82.89	81.84	88.5	87.64	91.47	84.16	86.75	93.11	101.49
Cerebrovascular diseases	46.66	47.53	48.46	50.13	47.60	50.37	49.90	48.85	51.6	50.13	51.91	53.24
Diabetes	39.15	39.88	40.43	42.07	40.62	42.35	41.79	39.75	42.36	43.72	48.79	52.7
Chronic liver disease and cirrhosis	22.22	21.38	20.75	21.20	19.98	20.15	19.33	18.30	17.97	16.81	17.32	17.61
Hypertensive diseases	19.97	21.43	21.56	23.33	23.59	25.01	25.78	25.41	26.51	28.44	33.60	37.39
Nephritis, nephrotic syndrome, and nephrosis	18.83	18.59	19.23	20.80	20.30	22.22	22.84	23.42	21.40	21.61	23.32	24.93

**TABLE 2:** Standardized annual mortality rates (per 100,000 persons) due to leading non-communicable diseases (Taiwan, 2011–2022). (The Impact of the COVID-19 Pandemicon Mortality Rates From Non-Communicable Chronic Diseases in Taiwan: An InterventionalTimeSeriesStudy.Taiwan. 2011-2022).

Year	Malignant tumor		Heart diseases		Cerebrovascular diseases		Diabetes		Chronic liver disease and cirrhosis		Hypertensive diseases		Nephritis, nephrotic syndrome, and nephrosis	
	Rate	Change (%)	Rate	Change (%)	Rate	Change (%)	Rate	Change (%)	Rate	Change (%)	Rate	Change (%)	Rate	Change (%)
2011	132.94	-	48.57	-	31.77	-	27.18	-	16.57	-	13.17	-	12.81	-
2012	132.06	−0.66%	48.58	0.02%	31.24	−1.67%	26.76	−1.55%	15.63	−5.67%	13.54	2.81%	12.27	−4.22%
2013	131.17	−0.68%	48.34	−0.49%	30.69	−1.76%	26.11	−2.43%	14.8	−5.31%	13.11	−3.18%	12.11	−1.30%
2014	130.87	−0.22%	50.95	5.40%	30.81	0.39%	26.32	0.80%	14.82	0.14%	13.7	4.50%	12.7	4.87%
2015	128.73	−1.64%	48.75	−4.32%	28.3	−8.15%	24.6	−6.53%	13.66	−7.83%	13.41	−2.12%	11.97	−5.75%
2016	127.47	−0.98%	51.05	4.72%	29	2.47%	24.8	0.81%	13.42	−1.76%	13.72	2.31%	12.63	5.51%
2017	124.13	−2.62%	49.14	−3.74%	27.87	−3.90%	23.77	−4.15%	12.67	−5.59%	13.6	−0.87%	12.56	−0.55%
2018	122.47	−1.34%	49.51	0.75%	26.47	−5.02%	21.81	−8.25%	11.65	−8.05%	13.02	−4.26%	12.47	−0.72%
2019	122.05	−0.34%	44.24	−10.64%	27.07	2.27%	22.54	3.35%	11.22	−3.69%	13.19	1.31%	10.91	−12.51%
2020	118.01	−3.31%	44.4	0.36%	25.59	−5.47%	22.3	−1.06%	10.34	−7.84%	13.7	3.87%	10.66	−2.29%
2021	118.87	0.73%	46.29	4.26%	25.52	−0.27%	24.11	8.12%	10.4	0.58%	15.52	13.28%	11.11	4.22%
2022	116.68	−1.84%	48.58	4.95%	25.45	−0.27%	25.04	3.86%	10.43	0.29%	16.65	7.28%	11.49	3.42%

Table 3 presents the pandemic’s effects on these non-communicable disease mortality rates (per 100,000 persons) in Taiwan. The model chosen for the analyzed variables of the intervention time series and the related parameters is presented. The most optimal models were chosen according to the methods previously described. All models showed a good level of fit according to Box-Ljung Q statistics. Statistical intervention or non-intervention models identified with monthly mortality as dependent variables showed significant (p < 0.001) intervention effects for heart diseases, cerebrovascular diseases, diabetes, and hypertension from 2011 to 2022. Heart diseases, diabetes, and hypertension saw a significant increase of 4.3%, 8.2%, and 13.4% in 2021 monthly mortality rates, continuing until the study period’s end. Conversely, the pandemic led to a 0.22% monthly decrease in cerebrovascular disease mortality.

**TABLE 3:** Effects of the intervention on standardized monthly mortality rates due to non-communicable diseases (Taiwan, 2011–2022). (The Impact of the COVID-19 Pandemicon Mortality Rates From Non-Communicable Chronic Diseases in Taiwan: An InterventionalTimeSeriesStudy.Taiwan. 2011-2022).

	Effect of intervention					SARIMA ${\left(p,d,q\right)\;\left(P,D,Q\right)}_{\left[S\right]}$ ^e^	Box–Ljung Q-statistics (df^a^)
Diseases	ω ^b^ (SE)	t-value	p-value	Mean before intervention	Change (%)^c^	Trend of intervention^d^	p-value
Malignant tumor	0.0994 (0.0772)	1.287	0.1981	9.8268	0.76	(1,0,0) (2,1,0)_12_ -	16.089 (19) 0.6513
Heart diseases	0.7407 (0.2195)	3.375	0.0007	3.7000	4.3	(1,0,2) (1,1,1)_12_ Gradual, temporary	13.138 (15) 0.5917
Cerebrovascular diseases	0.3741 (0.1145)	3.2687	0.0011	2.1324	−0.22	(1,0,0) (0,1,1)_12_ Gradual, temporary	22.904 (17) 0.1524
Diabetes	0.4567 (0.1081)	4.2258	<0.0001	1.8579	8.2	(2,0,0) (1,1,1)_12_ Gradual, temporary	22.774 (14) 0.06405
Chronic liver disease and cirrhosis	0.0371 (0.0478)	0.7773	0.437	0.862	0.56	(1,1,1) (2,0,0)_12_ -	14.916 (8) 0.06081
Hypertensive diseases	0.3746 (0.0504)	7.4375	<0.0001	1.1421	13.4	(1,0,1) (1,1,2)_12_ Abrupt, temporary	24.75 (17) 0.1005
Nephritis, nephrotic syndrome and nephrosis	0.0267 (0.0388)	0.6885	0.4911	0.8875	4.33	(1,0,2) (0,1,1)_12_ -	31.893 (21) 0.06

^a^ Degree of freedom.

^b^ Differences of standardized mortality rate of leading non-communicable diseases were estimated with a monthly autoregressive integrated moving average model.

^c^ Percent changes were based on the average of the 12 months immediately prior to and after the intervention.

^d^ Discriminated by intervention models.

^e^ Seasons.

## Discussion

Non-communicable chronic disease mortality rates have shown a downward trend in several countries in recent decades. However, since January 2021, we observed an upward trend in mortality rates during the COVID-19 pandemic, particularly for hypertensive diseases, heart diseases, and diabetes. A U.S. survey found that from March to April 2020, the annual percentage change in deaths increased by 4.9% for heart disease and 6.5% for diabetes [29, 31]. This may reflect patients’ fears of infection or hospital visits, leading to discontinued medical monitoring and worsening chronic health problems, posing a greater threat to patients needing long-term treatment [22, 32]. Initially, seniors and those of high socio-economic status reacted strongest, but post-lockdown, socio-economically disadvantaged communities had fewer medical visits. Neighborhoods with more females and young people showed larger decreases in medical visits throughout the periods. Disruptions varied across medical specialties, especially in cardiology [33]. Health promotion is crucial to reduce excess deaths among patients with fragile health and related metabolic conditions during the pandemic.

The rise in all-cause mortality rates during the COVID-19 pandemic highlights the impact of overloaded health systems [11]. Non-pharmaceutical prevention measures also limited healthcare accessibility. A global survey reported that 59% of countries had restricted outpatient services, with 12% completely interrupted; in Europe, 79% of countries reported interrupted rehabilitation services [34, 35]. An Italian study found hospitalizations for non-communicable diseases in 2020 decreased by about one-third compared to 2019 [36]. A UK study reported significant decreases in cancer screening, diagnosis, and referral and the suspension of national cancer screening programs [37]. Hospitalizations for acute coronary artery disease also dropped, potentially increasing deaths and long-term myocardial infarction complications [38]. In the U.S., 92% of vascular surgeries were canceled in April 2020 [39]. An estimated 28 million elective surgeries were canceled or delayed worldwide in 2020 due to COVID-19 control measures [40]. A Canadian study reported increased hospitalization deaths during the COVID-19 wave, with excess deaths due to respiratory and circulatory disorders and cancer [41].

A study from Taiwan found that people voluntarily reduced their demand for healthcare, even without mobility restrictions or supply-side constraints. As a result, outpatient visits and inpatient hospitalizations decreased temporarily during the first wave of the pandemic in 2020. During the alert period, the CECC separated COVID-19 patients into different wards and improved the referral mechanism for severe and mild cases. Outpatient and hospitalization claims decreased by 18.1% and 26%, respectively, in the first half of 2021 compared to the previous year [42]. Emergency department volume also decreased in 2021 [43]. A review showed that COVID-19 negatively impacted Taiwan’s healthcare system, including emergency services for out-of-hospital cardiac arrests [44]. Thus, the reduction in medical service volume might have raised severe concerns for needy people.

Nonetheless, it is important to note that Taiwan’s first large-scale outbreak occurred later than in many other countries, potentially altering the timing and magnitude of these service disruptions. Future research should consider how this delayed onset may have influenced patterns of healthcare utilization and mortality outcomes.

This study found a slight decline in cerebrovascular disease mortality since January 2021. Unlike chronic conditions such as hypertension, heart disease, and diabetes—whose management often requires consistent outpatient follow-up—acute cerebrovascular events (e.g., strokes) may prompt urgent care-seeking even during the pandemic, potentially mitigating mortality increases [45]. No significant impacts on mortality from malignant tumors, renal diseases, or liver diseases were observed during the pandemic. NHIA statistics show that hematology, oncology, rheumatology, and immunology increased by 7.9% and 1.5% in the first half of 2021. During the Level-3 Alert period, the NHIA adjusted measures to mitigate the impact on medical care, including expanding home medical care and telemedicine, allowing community pharmacies to deliver medicine, and allowing one-time collection of medications for chronic diseases [42]. It is worth exploring whether these measures alleviated the impact of reduced in-person visits on chronic disease mortality.

Our study suggests that reducing specific medical services during a pandemic may affect mortality from certain causes. This supports recommendations to avoid reducing healthcare services whenever possible and to increase hospital capacity if needed [46, 47]. Planning should adapt to rapidly evolving pandemic needs while ensuring safe and timely access to care. Individuals’ behaviors can directly or indirectly impact non-communicable disease outcomes. A review found that during the COVID-19 pandemic, adverse health effects arose from changes in cardiovascular risk factors due to control measures, such as increased food intake, prolonged sitting, reduced activity, smoking, drinking, weight gain, and changes in blood pressure and lipids [48]. A US study found increased odds of metabolic conditions among adults who smoked or had anxiety/depression during the pandemic [49]. Further studies and proper public health resource allocation to address these conditions were recommended.

This study used an interventional time-series model to evaluate long-term trends in a nationwide population, correcting long-term effects better than segmental time difference comparisons. This empirical evidence demonstrates the temporality of impacts, enhancing research inference credibility. However, several limitations remain. Firstly, our findings may not be generalizable internationally, as the study used only Taiwan claim data. Countries with different healthcare infrastructures or distinct pandemic timelines may experience different patterns of chronic disease mortality, thus limiting the direct applicability of our findings beyond Taiwan. Secondly, excess mortality from non-communicable diseases may be misestimated due to misclassification of death causes. Despite excluding causes directly associated with COVID-19, it was challenging to determine if deaths resulted from acute or chronic conditions worsened by prior COVID-19. However, the study focused on mortality impact rather than cause identification, so misclassification may not alter findings. Nevertheless, misclassification may also account for some of the observed increases in mortality from hypertension, heart disease, and diabetes.

In Taiwan, the official cause of death is determined under a standardized process involving physicians, local authorities, and—for non-natural or unclear cases—law enforcement and forensic experts. Death certificates follow ICD-10 guidelines, identifying the root (underlying) cause as well as any contributing conditions. A COVID-19 death generally requires a PCR-confirmed SARS-CoV-2 infection, along with physician assessment in line with Taiwan CDC guidelines. Although this system strives for accuracy, overlapping comorbidities (e.g., COVID-19 and chronic conditions) or ambiguous clinical findings can still lead to COVID-19 cases being recorded under these chronic diseases, thereby inflating their mortality counts. Therefore, the observed increase in hypertension, heart disease, or diabetes mortality could partly reflect incomplete or inaccurate labeling rather than purely reflecting disruptions in healthcare access or behavior changes.

Lastly, without an explicit intervention date for annual comparisons, the model was based on January 2021. Observing the one-step-ahead forecast in Figure 1, effects may have begun as early as early 2020. Future studies should explore correlations between death and hospitalization rates and individual health behaviors before, during, and after the epidemic. These investigations can provide further empirical evidence linking changes in death rates to medical service factors.

### Conclusion

This study found an upward trend in mortality from non-communicable diseases, such as hypertension, heart diseases, and diabetes, during the COVID-19 pandemic. The empirical evidence demonstrated the trend and impact timeline, enhancing research inference credibility and value. Health promotion and medical resource allocation were crucial for patients with disadvantaged health and sociodemographic factors and related metabolic conditions during the pandemic. Future studies should explore correlations between trends in death and hospitalization rates and changes in adverse health behaviors during pre- and post-pandemic periods.
